# Supplementary material for: Complex‐centric proteome profiling by SEC‐SWATH‐MS
Source: Mol Syst Biol. 2019 Jan 14;15(1):e8438. doi: 10.15252/msb.20188438 (PMC6346213; doi:10.15252/msb.20188438)
Supplement: Supplementary file 8 — Dataset EV7 [file MSB-15-e8438-s008.zip › feature_plots_string/Q9NXX6.pdf]

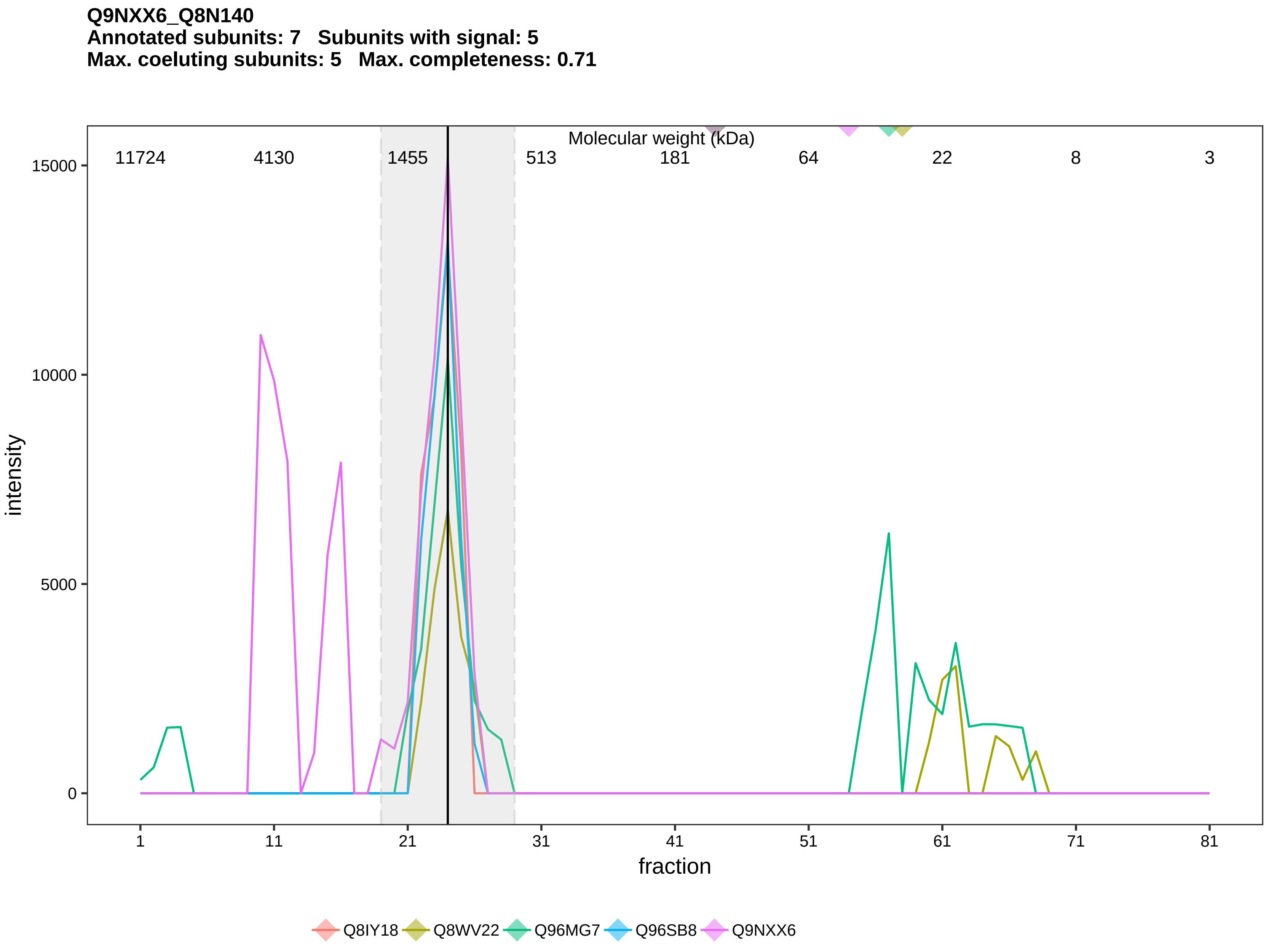
Q9NXX6_Q8N140
Annotated subunits: 7 Subunits with signal: 5
Max. coeluting subunits: 5 Max. completeness: 0.71
Molecular weight (kDa)
11724
4130
1455
513
181
64
22
8
3
intensity
15000
10000
5000
0
1
11
21
31
41
51
61
71
81
fraction
Q8IY18
Q8WV22
Q96MG7
Q96SB8
Q9NXX6
